# Adolescent Socioeconomic and School-Based Social Status, Smoking, and Drinking

**DOI:** 10.1016/j.jadohealth.2015.03.020

**Published:** 2015-07

**Authors:** Helen Sweeting, Kate Hunt

**Affiliations:** MRC/CSO Social and Public Health Sciences Unit, University of Glasgow, Glasgow, United Kingdom

**Keywords:** Adolescent, Subjective social status, Socioeconomic status, School-based social status, Peer status, Smoking, Drinking, Psychological well-being, United Kingdom

## Abstract

**Purpose:**

Relationships between subjective social status (SSS) and health-risk behaviors have received less attention than those between SSS and health. Inconsistent associations between school-based SSS and smoking or drinking might be because it is a single measure reflecting several status dimensions. We investigated how adolescent smoking and drinking are associated with “objective” socioeconomic status (SES), subjective SES, and three dimensions of school-based SSS.

**Methods:**

Scottish 13–15 years-olds (N = 2,503) completed questionnaires in school-based surveys, providing information on: “objective” SES (residential deprivation, family affluence); subjective SES (MacArthur Scale youth version); and three school-based SSS dimensions (“SSS-peer”, “SSS-scholastic” and “SSS-sports”). We examined associations between each status measure and smoking (ever and weekly) and drinking (ever and usually five or more drinks) and investigated variations according to gender and age.

**Results:**

Smoking and heavier drinking were positively associated with residential deprivation; associations with family affluence and subjective SES were weak or nonexistent. Both substances were related to each school-based SSS measure, and these associations were equally strong or stronger than those with deprivation. Although SSS-peer was positively associated with both smoking and (especially heavier) drinking, SSS-scholastic and SSS-sports were negatively associated with both substances. There were no gender differences in the associations and few according to age.

**Conclusions:**

Subjective school-based status has stronger associations with adolescent smoking and drinking than “objective” or subjective SES. However, different dimensions of school-based status relate to adolescent smoking and drinking in opposing directions, meaning one measure based on several dimensions might show inconsistent relationships with adolescent substance use.

**Implications and Contribution**

A single, “overall” school-based status measure based on several dimensions (e.g., “friends,” “grades,” “sports”) might show inconsistent relationships with adolescent substance use because these different dimensions are associated with smoking and drinking in different directions. The methodological implication is that school-based status measures should include a range of dimensions.

There is a vast literature relating to associations between adolescent smoking and drinking and socioeconomic status (SES) as represented by “objective” variables, such as parental occupation, income, education, or neighborhood resources. Higher smoking rates are generally found among low SES adolescents [1], with the strongest SES effects in early adolescence [2]. However, studies variously find either no relationship, positive, or negative associations between SES and adolescent drinking [1]. There is also evidence that associations with SES differ according to the smoking/drinking measure employed: heavier smoking and alcohol misuse are more likely among low SES adolescents; occasional smoking; and experimental alcohol among high SES adolescents [3,4].

In contrast to more “objective” status measures, subjective social status (SSS) has been defined as a person's sense of place within a hierarchy and is usually measured by asking respondents where they would place themselves on a picture of a ladder. The most common adult version asks respondents to mark where they would place themselves on a ladder “representing where people stand in [country name]”. There is also a second, “community” ladder, which asks respondents to define community “in whatever way is most meaningful to you” [5]. SSS scales for adolescents have asked them where “your family would be on this ladder” (SSS-society) and, on the basis that school is their most salient community, where they would place themselves on a ladder where the highest rung represents “the people in your school with the most respect, the highest grades, and the highest standing” [6].

Despite a growing literature on associations between SSS and health or well-being (e.g., [7,8]), the relationship between SSS and health-risk behaviors has received much less attention. We are aware of only two studies of subjective SES as represented by the SSS-society ladder and adolescent smoking or drinking. Among U.S. 12–17 year olds, “perceived SES” was inversely associated with ever smoking and with smoking uptake [9]. Among disadvantaged Mexican 12–22 year olds, “society SSS” was inversely related to both smoking and drinking, even after adjustment for “community SSS” and sociodemographic characteristics [10]. Others have included different subjective SES measures with different results. For example, among U.S. 13 year olds, smoking was not associated with any of several perceived SES measures (family's ability to afford basic necessities, perceived wealth relative to others, perceived wealth relative to last year) [11]. Similarly, self-assessed SES was not associated with smoking among Hungarian 14–21 year olds but was related to drinking (highest levels among those reporting themselves to be “upper class”), even after the adjustment for several “objective” SES measures [12]. A meta-analysis concluded subjective SES was not associated with adolescent substance-related health behaviors [7].

Two of these studies also examined associations between measures of SSS-community/school and adolescent smoking/drinking. In the study of US 12–17 year olds by Finkelstein et al. [9], “subjective school status” was inversely associated with smoking, both cross-sectionally and one year later. However, among disadvantaged Mexican 12–22 year olds, Ritterman et al. [10] found “community SSS” was positively related to both smoking and drinking in bivariate and adjusted analyses. In another study, of 11–13 year old Mexican Americans (which did not include subjective SES), Wilkinson et al. found SSS-school was inversely associated with experimental smoking [13] and drinking [14].

Finkelstein et al. [9], whose measure of SSS-school was based on “respect,” “grades,” and “standing,” suggest that their results arise because high SSS-school is associated with academic success (known to be inversely related to smoking), popularity (possibly protecting against smoking in schools with low smoking rates), and participation in activities that deter smoking. Ritterman et al. [10] suggest that because many in their Mexican sample did not attend school, their peer-based measure of SSS might have tapped popularity and social integration, but not academic achievement. Wilkinson et al. [10] used a measure based on “grades,” “friends,” and “sports.” They suggest that although adolescents “may see smoking as a way of obtaining more friends, it is less likely that they believe smoking will help them get better grades and be more athletic” [13] p347, and that reasons for adolescent drinking, such as to relieve anxiety or be cool, may be related to SSS [14].

It is clearly possible that different associations with smoking/drinking in these studies might have arisen from the different SSS-community/school measures used. Furthermore, the SSS-school measures reflect several status dimensions (“respect,” “standing,” “friends,” “grades,” “sports”) [9,13] which are not necessarily well correlated [15]. The wider literature, summarized in the following, provides evidence that popularity or social integration, scholastic achievement, and sporting status might not all be associated with smoking/drinking in the same way.

Sociometric studies have variously shown smoking to be more likely among “rejected and controversial” adolescents [16], those in dyadic friendships or “isolates” [17], but also those perceived as “popular,” occupying powerful network positions [18] and those whose popularity increases during middle childhood [19]. Peer-nominated sociability, self-confidence [20], and higher self-rated popularity have also been associated with adolescent smoking [21]. Some studies have found that adolescent smoking is positively associated with peer-based time [22], wheras others have not [23]. Clearly, smoking is associated with peer relationships in complex ways [24,25], and indeed some [26,27], but not all [24], studies suggest higher smoking rates at both ends of the social hierarchy, with nonsmokers tending to be “middle” pupils. Despite some evidence that adolescents associate the same traits with both smoking and drinking [20], other studies suggest that peer processes operate in different ways for the two substances [17,28], with adolescent drinking more clearly associated with self-confidence, power, sociability, popularity (sociometric, perceived, and self-report), and fun [17,18,20–23].

Low school engagement, misbehavior, and poor academic achievement are consistently associated with smoking, but less consistently with drinking [21,22,29]. Similarly, low physical activity and sports achievement are associated with smoking although the relationship between physical activity/sports and drinking is less clear [22,29,30]. Thus, we might hypothesize that a peers/popularity/power-based SSS-school measure would show a U-shaped relationship with smoking and a positive relationship with drinking, whereas measures representing scholastic achievement or sporting status might show an inverse relationship with smoking and possibly also with drinking.

As an additional layer of complexity, it is possible that associations between any school-based status measure and adolescent smoking/drinking might differ according to gender and/or age. Although social relationships may be particularly important for females, males' greater emphasis on sports [26,28] mean we might expect gender differences in associations between smoking/drinking and SSS-school measures based on peers/popularity/power (stronger among females) and sports (stronger among males). However, some studies have found no gender differences in associations between adolescent sociometric measures, academic achievement or sports-based time-use, and smoking/drinking [17,19,22]. It is also possible that as smoking/drinking are more normative among older adolescents, their associations with school-based status measures might change with age [19,20,24].

Against this background, using data from Scottish adolescents, we address the following questions:

• How are adolescent smoking and drinking associated with “objective” SES, SSS-society (henceforth subjective SES), and SSS-school (henceforth subjective school-based status)?
• Do associations with social status differ for smoking compared with drinking?
• Do associations with social status differ for lighter versus heavier (i.e., less vs. more deviant) levels of smoking and drinking?
• Are different dimensions of subjective school-based status associated with smoking/drinking in the same way?
• Do associations between measures of subjective school-based status and smoking/drinking vary according to gender or age?

## Methods

### Sample

Data are from a study of school pupils, surveyed in 2010, with follow-up in 2011. The sample comprised seven schools with different socioeconomic catchments (indicated by proportions receiving free school meals) from two urban and semi-rural areas in Scotland's central belt. All pupils in selected year groups were invited to participate via letters to parents including parental opt-out consents. Pupils separately received information and gave written consent before participation. The study was approved by the relevant Glasgow University Ethics Committee, local education authorities and schools.

The baseline sample comprised 2,937 pupils in Secondary 1, S2, and S3 year groups, representing 92% of the 3,189 eligible. Levels of nonconsent were very low (11 parents and 15 pupils); thus, almost all nonresponders were absentees. A total of 2,503 pupils of the baseline sample also participated at follow-up in 2011, when they were in the S2, S3, and S4 year groups (aged 13–15 years). These analyses use data obtained in 2011 with the addition of family affluence, obtained in 2010 (this scale was not included in the 2011 survey). This restricts the sample to those participating at both dates.

Pupils completed questionnaires in examination-type conditions during school-based sessions, led by researchers and survey assistants [31].

### Measures

#### “Objective” socioeconomic status

“Objective” SES measures included residential deprivation, represented via the Scottish Index of Multiple Deprivation (SIMD-2009). This identifies concentrations of multiple deprivation across Scotland by assigning a score to small areas based on postcodes, derived from national indicators covering seven domains (income, employment, health, education, service access, housing, crime). This methodology is widely accepted; similar methodologies are used across Great Britain and Northern Ireland [32]. Scottish Index of Multiple Deprivation deciles are ranked 1 (most deprived) to 10 and were available for 77% of our analysis sample. We also included the Family Affluence Scale, range 0–7, based on the number of family cars, vans/trucks; family computers; past year family holidays; and own bedroom. This has been found to be reliable (pupils' and parents' reports on component items agree) and sensitive in differentiating between-country levels of affluence [33].

#### Subjective socioeconomic status

Subjective SES was measured via the youth version of the MacArthur Scale of Subjective Social Status [6], with some wording adapted for Scottish adolescents. The 10-rung ladder image included the instructions: “Imagine this ladder shows how Scottish society is set up. Now think about your family. Please tell us where you think your family would be on this ladder.” The top rung was labeled “the best off people in Scotland–they have the most money, the most education, and the jobs that bring most respect” and the bottom rung “the worst-off people in Scotland–they have the least money, not much education and no job, or a job that no-one wants or respects.”

#### Subjective school-based status

Subjective school-based status measures were based on previous work and derived from seven further 10-rung ladder pictures, with the instructions “Imagine these ladders show where people fit in your year group. Where would you put yourself?” [31,34] These ladders asked pupils to rate themselves on the following: (1) popularity; (2) school performance; (3) being powerful; (4) being a troublemaker; (5) attractiveness or stylishness; (6) being respected; and (7) being sporty, compared with the rest of the year group. Factor analysis suggested three dimensions which we describe as “SSS-peer” (“popular,” “powerful,” “respected,” “attractive/stylish,” “troublemaker”), “SSS-scholastic” (“doing well at school”; not a “troublemaker”), and “SSS-sports” (“sporty”). Previous analyses found each dimension had unique relationships with variables representing more “objective” and/or self-report behavioral measures [34]. Analyses reported here used the three factor scores. Note our questionnaire did not include a single SSS-school measure based on multiple dimensions as used in previous studies of associations between measures of SSS-school and adolescent smoking/drinking [9,13,14].

#### Smoking and drinking

Responses to the questions “how many cigarettes have you smoked in your life” were dichotomized to ever smokers versus never smokers, and “how often do you smoke at present,” to weekly smokers versus less frequent or nonsmokers. Similarly, “have you ever drunk any alcohol, even just a sip” was dichotomized to ever drinkers versus never drinkers and “how many drinks do you usually drink at one time” to usually five or more drinks versus fewer or nondrinkers.

### Analyses

Associations between status and smoking/drinking were examined via cross-tabulation and logistic regression (bivariate and mutually adjusted analyses). To investigate whether both high and low status were associated with smoking/drinking, each status measure (including the factor scores representing school-based status) was collapsed into three categories representing (approximately) the lowest 25%, mid 50%, and highest 25% of the sample. An additional “missing” category was included for residential deprivation because this comprised 23% of the sample (results relating to this not discussed further). Tests of interactions with gender and year group were conducted to identify any between-group differences in the status-smoking/drinking associations.

Our baseline sample showed similar family affluence levels to a Scotland-wide school-based survey, conducted at the same time [35]. Analyses included probabilistic weights to compensate for differential attrition at follow-up and accounted for clustering of data within school classes. Analyses were conducted on those with complete data on all relevant variables (N = 2,346 for ever smoker; 2,345 for weekly smoker; 2,347 for ever drinker; 2,342 for usually five or more drinks).

## Results

Preliminary analyses examined relationships between status measures (Table 1). Subjective SES had a much stronger association with family affluence than that with residential deprivation. Of the school-based status measures, only SSS-scholastic was related to residential deprivation; it was also the only school-based status measure with similar-sized relationships with each SES measure. Subjective SES was strongly related to each school-based measure, particularly SSS-peer.

Table 2 reports the associations between both ever and weekly smoking, and gender, year group, and all status measures. Overall, 32% reported ever and 11% weekly smoking (Table 2, final row). Bivariate analyses (see column b) showed no significant gender differences in smoking, but as would be expected, strong associations with age, the likelihood of both ever and weekly smoking being around three times higher in the S4, compared with S2 year group. Both measures, particularly weekly smoking, were also strongly associated with residential deprivation; 4% from low and 17% from high deprivation areas reported weekly smoking. However, relationships with both family affluence and subjective SES were much weaker. In contrast, all three school-based status measures were strongly associated with smoking. Thus, rates of ever smoking for those high versus low on these measures were: 46% versus 26% (SSS-peer); 12% versus 55% (SSS-scholastic); and 24% versus 39% (SSS-sports). In mutually adjusted models (column c), associations with age remained largely unchanged, but those with SES all weakened. Thus, although both ever and weekly smoking remained strongly associated with residential deprivation, relationships with family affluence and subjective SES reduced to nonsignificance. Associations between the school-based status measures and smoking were generally very similar in the bivariate and adjusted models.

Table 3 shows the results of identical analyses in respect of ever drinking; 92% of the overall sample were ever drinkers, and 20% reported usually five or more drinks at a time (final row). Again, bivariate analyses showed no significant gender differences in either measure, but increases with age, particularly in usually consuming five or more drinks when drinking (reported by 10% of the S2 and 33% of the S4 year groups). Although ever drinker was not associated with either “objective” or subjective SES, reporting usually consuming five or more drinks was greater among those from high (26%) compared with low (12%) deprivation areas and those reporting low (24%) compared with high (18%) subjective SES. Both drinking measures were associated positively with SSS-peer but negatively with SSS-scholastic. Usually consuming five or more drinks was also negatively associated with SSS-sports. Mutual adjustment had little impact on the relationships between either drinking measure and SSS-peer or SSS-scholastic. Associations between usually consuming five or more drinks and both deprivation and SSS-sports reduced slightly after adjustment, but remained strong.

Given evidence that adolescents link being a troublemaker with substance use [26], it is possible that this might have led smoker and/or drinker respondents to place themselves high on the troublemaker ladder. Hence, inclusion of troublemaker within our SSS-peer factor might have produced the relationship between SSS-peer and smoking/drinking. We therefore conducted a sensitivity analysis, based on a variable derived by summing responses to the “popular,” “powerful,” “respected,” and “attractive or stylish” ladders. This was also strongly associated with both smoking and drinking: rates among those low compared with high status on this measure were 15% versus 9% (weekly smoking) and 31% versus 14% (usually five or more drinks; Supplementary Table 1).

Additional analyses showed no significant (*p* < .05) interactions between any of the school-based status measures and gender. The few interactions with year group included stronger associations among the youngest pupils between deprivation and both ever smoker and usually five or more drinks, with a similar trend for weekly smoker and also between SSS-peer and both weekly smoking and usually five or more drinks. Associations between SSS-scholastic and both weekly smoking and usually five or more drinks tended to be somewhat weaker among younger pupils (Supplementary Tables 2 and 3).

## Discussion

Although the main focus of this article was relationships between adolescent smoking and drinking and a range of “objective” and SSS measures, preliminary analyses examined relationships between the status measures. We discuss these first. As previously reported, strong relationships between subjective SES and family affluence but not residential deprivation in this sample suggest adolescents base subjective SES assessments on household/material rather than area-based characteristics [31]. Inconsistent associations between different school-based status and SES measures were also found in another sample [34]. Strong associations between subjective SES and subjective school-based status could be attributed to shared method variance, both likely influenced by personal characteristics such as self-esteem.

In relation to associations between smoking/drinking and our two “objective” SES measures, both smoking and heavier levels of drinking were related to residential deprivation, but only smoking showed an association with family affluence, and only in bivariate analyses. The “objective” measures represent different aspects of SES, with results suggesting that within-household material deprivation impacts less on adolescent smoking and drinking than local area deprivation.

Subjective SES showed significant or borderline associations with smoking and heavier levels of drinking. For smoking, but not drinking, associations with subjective SES reduced after adjustment for the other status measures, possibly because smoking had stronger relationships with “objective” SES. It has been suggested that in adolescence, subjective SES may reflect the influence of modern consumer culture (emphasizing possessions, brands and/or appearance) and “objective” SES [8]; previous analyses have shown associations between indicators of consumerism and both smoking and drinking this sample [35].

Both smoking and drinking were related to each measure of subjective school-based status in bivariate and adjusted analyses, and these associations were equally strong or much stronger than those with residential deprivation. This is consistent with suggestions that school-based status reflects immediate, micro-level processes [36], potentially of greater relevance to adolescents than macro-level status measures [8,15] as represented here by either “objective” or subjective SES. Smoking and drinking showed similar associations with each status measure. Relationships were stronger for heavier drinking levels (usually consuming five or more drinks) than those for the lighter measure (ever drinker). The two smoking measures showed less variation. These differences reflect the normative nature of ever drinking, compared with heavier drinking or any level of smoking, among this sample. However, despite the fact that 92% reported ever drinking, this measure was still strongly associated with SSS-peer and SSS-scholastic, suggesting the importance of these dimensions.

In explaining these relationships, we begin with SSS-peer and previous suggestions that this measure may represent an amalgam of the two types of popular pupils (liked and visible) [34]. Although some previous literature [26,27] suggests that both high and low peer status are associated with smoking/drinking, we found no evidence for a U-shaped relationship. SSS-peer was positively associated with both smoking and drinking, especially heavier drinking. A small part of this relationship could be attributed to the inclusion of “troublemaker” within the measure; adolescents may define themselves as “troublemakers” because of smoking and/or drinking. However, our results agree with other studies linking adolescent smoking and drinking to characteristics such as popularity, power, and sociability [17–23,37]. Popular pupils might have more smoking or drinking opportunities or engage in these behaviors to maintain or demonstrate their high status [38]. Much higher levels of smoking and drinking among low, compared with high SSS-scholastic pupils may reflect academically unsuccessful adolescents engaging in these behaviors as alternative ways to achieve positive self-image and social success. There is evidence that academic difficulties tend to predict substance use, rather than vice versa [39]. Scholastic difficulties and substance use might also have common causes, including poor impulse control or less effective parenting [16]. One possible reason for higher levels of smoking and drinking among lower SSS-sports pupils is that sports require physical fitness, so smoking is unlikely to be an attractive option for sporty adolescents [30].

Importantly, both substances were associated positively with SSS-peer but negatively with SSS-scholastic and SSS-sports. The strength of association also differed between outcome measures. Smoking was most strongly associated with SSS-scholastic, consistent with it being a marker of adolescent rebelliousness and low achievement motivation [40]. Drinking was least strongly associated with SSS-sports, perhaps because reduced drinking resulting from participating in activities associated with fitness and adult supervision is slightly offset by drinking among sports-related social networks [22,29,30]. Crucially, these results mean a single SSS-school measure, based on dimensions such as “respect,” “standing,” “friends,” “grades,” and “sports,” might show inconsistent relationships with adolescent smoking/drinking. If respondents focus on the dimension most significant to themselves (which is likely to be that on which they would rank themselves highest), some might choose one which happens to be positively associated with smoking/drinking, whereas others choose one negatively associated. Future studies could examine this by measuring SSS-school via both a single “overall” ladder and ladders representing different status dimensions.

We found no gender differences in the associations and few according to age. However, consistent with others [2], there were stronger associations with “objective” SES among younger pupils for all except the arguably normative behavior of ever having had an alcoholic drink. We also hypothesized the more normative nature of smoking/drinking among older adolescents might mean associations with school-based status changed with age [19,20,24]. There was some weak evidence of this in respect of the two heavier measures (weekly smoking and usually five or more drinks); SSS-peer was most strongly associated with these measures among the youngest pupils. High SSS-peer pupils tend to come from well-resourced families, receive most pocket money, have most friends, and be rated by research interviewers as more physically attractive and mature [34]. It is unsurprising that such characteristics were most strongly associated with smoking and drinking among the youngest pupils, for whom these behaviors were least normative. In contrast, SSS-scholastic was least strongly related to these smoking/drinking measures among younger pupils, perhaps reflecting schools' increasing emphasis on academic achievement with age (most Scottish pupils take their first national qualifications in S4); SSS-scholastic matters less among the youngest pupils.

Our analysis is limited by the fact that it is based on cross-sectional data, making it impossible to confidently infer direction of causality. Nevertheless, it adds to the small body of work on SSS and health-risk behaviors and has substantive, practical, and methodological implications. Substantively, our results are consistent with suggestions that health-related behavior may be one mechanism in the association between peer status and morbidity [36]. Practically, they suggest the need for those working in schools to acknowledge the importance of school-based social status and their own potential role in contributing to or facilitating pupils' social interactions and peer relationships. Methodologically, and in the context of calls for increasing use of SSS measures in studies of adolescents [7], they highlight the need to consider how different dimensions of adolescent school-based status might relate to adolescent smoking and drinking in opposing directions and suggest that SSS-school may not be adequately represented by a single, overall measure.

## Figures and Tables

**Table 1:** Relationships between status measures—cross-tabulations showing column percentages, chi-square (and significance)

	Residential deprivation				Family affluence scale				Subjective socioeconomic status			
	High status (low deprivation)	Medium status	Low status (high deprivation)	Chi-square (significance)	High status (high affluence)	Medium status	Low status (low affluence)	Chi-square (significance)	High status	Medium status	Low status	Chi-square (significance)
Residential deprivation												
High status (low deprivation)					42.2	31.3	17.2		31.9	34.2	22.3	
Medium status					48.0	50.0	52.2	92.5	50.6	48.2	53.7	19.6
Low status (high deprivation)					9.8	18.7	30.6	<.001	17.5	17.7	24.0	.001
Family Affluence Scale												
High status (high affluence)									34.6	23.9	14.9	
Medium status									49.6	54.4	49.5	94.1
Low status (low affluence)									15.8	21.7	35.6	<.001
SSS-peer												
High status	22.3	24.0	26.2		29.4	24.9	20.2		37.4	22.6	13.6	
Medium status	51.3	51.6	49.0	2.3	50.1	50.9	48.4	23.7	47.8	53.0	45.6	144.9
Low status	26.4	24.4	24.8	.686	20.5	24.3	31.4	<.001	14.8	24.3	40.8	<.001
SSS-scholastic												
High status	31.6	26.7	20.7		30.7	25.3	17.8		30.3	24.3	19.0	
Medium status	52.6	50.6	47.8	34.4	47.4	51.7	50.7	34.0	49.5	51.3	49.0	29.8
Low status	15.8	22.7	31.5	<.001	21.9	23.1	31.6	<.001	20.3	24.3	32.0	<.001
SSS-sports												
High status	28.8	23.3	21.6		25.6	26.2	21.9		30.3	24.9	18.1	
Medium status	47.5	51.6	53.1	8.0	50.9	49.4	49.7	6.2	50.2	50.0	48.8	35.9
Low status	23.7	25.1	25.4	.091	23.4	24.5	28.4	.182	19.5	25.1	33.1	<.001

SSS = subjective social status.

**Table 2:** Ever and weekly smoker according to gender, school year group, and status measures: (a) numbers (and row percentages); (b) unadjusted ORs (and 95% CIs); and (c) mutually AORs (and 95% CIs) for model including gender, year group, and all status measures

	Ever smoker						Weekly smoker					
	Total N analyzed	(a) *% ever smokers*	(b) Unadjusted ORs		(c) Mutually adjusted ORs		Total N analyzed	(a) *% weekly smokers*	(b) Unadjusted ORs		(c) Mutually adjusted ORs	
			OR (95% CI)	*t*	AOR (95% CI)	*t*			OR (95% CI)	*t*	AOR (95% CI)	*t*
Gender												
Males	1,197	*31.6*	1.00		1.00		1,196	*11.2*	1.00		1.00	
Females	1,149	*32.0*	1.04 (.85–1.27)	*.4*	1.16 (.92–1.45)	*1.3*	1,149	*10.2*	.92 (.68–1.24)	*−.5*	.93 (.67–1.29)	*−.5*
Year group												
S2 (age 13 years)	807	22.2	1.00		1.00		807	*5.8*	1.00		1.00	
S3 (age 14 years)	786	30.8	1.59 (1.20–2.10)	*3.2*	1.57 (1.17–2.12)	*3.0*	785	*10.2*	1.88 (1.30–2.74)	*3.3*	1.82 (1.25–2.65)	*3.2*
S4 (age 15 years)	753	43.2	2.78 (2.18–3.54)	*8.3*	2.93 (2.19–3.91)	*7.4*	753	*16.5*	3.30 (2.31–4.70)	*6.6*	3.35 (2.27–4.96)	*6.1*
Residential deprivation												
High status (low deprivation)	569	*19.0*	1.00		1.00		568	*4.4*	1.00		1.00	
Medium status	909	*31.2*	1.89 (1.41–2.52)	*4.4*	1.74 (1.29–2.34)	*3.7*	909	*9.9*	2.37 (1.44–3.90)	*3.4*	1.97 (1.20–3.25)	*2.7*
Low status (high deprivation)	343	*42.3*	3.09 (2.16–4.43)	*6.2*	2.51 (1.69–3.72)	*4.6*	343	*16.9*	4.55 (2.59–7.98)	*5.3*	3.17 (1.72–5.84)	*3.7*
Missing	525	*39.8*	2.87 (2.15–3.83)	*7.2*	2.20 (1.64–2.93)	*5.4*	525	*14.9*	3.95 (2.40–6.51)	*5.4*	2.56 (1.53–4.28)	*3.6*
Family Affluence Scale												
High status (high affluence)	588	*28.9*	1.00		1.00		588	*9.7*	1.00		1.00	
Medium status	1,223	*30.7*	1.04 (.84–1.30)	*.4*	.97 (.77–1.22)	*−.3*	1,222	*8.9*	.90 (.67–1.22)	*−.7*	.86 (.61–1.23)	*−.8*
Low status (low affluence)	535	*37.4*	1.44 (1.10–1.89)	*2.7*	1.06 (.80–1.41)	*.4*	535	*15.9*	1.82 (1.28–2.58)	*3.4*	1.37 (.91–2.05)	*1.5*
Subjective socioeconomic status												
High status	647	*28.1*	1.00		1.00		647	*9.6*	1.00		1.00	
Medium status	1,236	*31.6*	1.17 (.93–1.47)	*1.3*	1.12 (.85–1.47)	*.8*	1,235	*10.3*	1.09 (.81–1.48)	*.6*	.97 (.69–1.37)	*−.2*
Low status	463	*37.6*	1.50 (1.14–1.97)	*3.0*	1.27 (.91–1.78)	*1.4*	463	*13.4*	1.44 (.96–2.16)	*1.8*	1.06 (.64–1.73)	*.2*
SSS-peer												
High status	585	*45.5*	1.00		1.00		584	*17.5*	1.00		1.00	
Medium status	1,175	*28.1*	.47 (.37–.61)	*−6.0*	.48 (.36–.62)	*−5.5*	1,175	*9.1*	.49 (.36–.66)	*−4.6*	.53 (.39–.73)	*−3.9*
Low status	586	*25.6*	.41 (.31–.55)	*−6.0*	.34 (.24–.47)	*−6.2*	586	*7.2*	.38 (.25–.57)	*−4.7*	.35 (.22–.56)	*−4.4*
SSS-scholastic												
High status	585	*11.6*	1.00		1.00		585	*2.6*	1.00		1.00	
Medium status	1,182	*30.5*	3.39 (2.53–4.56)	*8.2*	3.50 (2.57–4.75)	*8.1*	1,182	*8.5*	3.38 (1.98–5.78)	*4.5*	3.17 (1.81–5.58)	*4.1*
Low status	579	*54.7*	9.53 (6.97–13.02)	*14.3*	9.24 (6.76–12.64)	*14.0*	578	*23.5*	11.70 (6.86–19.91)	*9.1*	9.77 (5.69–16.81)	*8.3*
SSS-sports												
High status	588	*23.8*	1.00		1.00		587	*5.8*	1.00		1.00	
Medium status	1,170	*32.0*	1.52 (1.23–1.87)	*3.9*	1.34 (1.05–1.71)	*2.4*	1,170	*11.3*	2.04 (1.37–3.03)	*3.5*	1.81 (1.19–2.74)	*2.8*
Low status	588	*39.3*	2.07 (1.60–2.67)	*5.7*	1.94 (1.48–2.54)	*4.9*	588	*14.5*	2.86 (1.76–4.66)	*4.3*	2.66 (1.64–4.30)	*4.0*
*N (% ever/weekly smokers)*	*2,346*	*31.8*					*2,346*	*10.7*				

OR = odds ratio; CI = confidence interval; AOR = adjusted odds ratio; SSS = subjective social status.

**Table 3:** Ever drinker and usually consume more than five drinks at a time according to gender, school year group, and status measures: (a) numbers (and row percentages); (b) unadjusted ORs (and 95% CIs); and (c) mutually AORs (and 95% CIs) for model including gender, year group and all status measures

	Ever drinker						Usually consumes five or more drinks					
	Total N analyzed	(a) *% ever drinkers*	(b) Unadjusted ORs		(c) Mutually adjusted ORs		Total N analyzed	(a) *% usually five or more drinks*	(b) Unadjusted ORs		(c) Mutually adjusted ORs	
			OR (95% CI)	*t*	AOR (95% CI)	*t*			OR (95% CI)	*t*	AOR (95% CI)	*t*
Gender												
Males	1,197	*91.7*	1.00		1.00		1,194	*19.8*	1.00		1.00	
Females	1,150	*92.2*	1.11 (.84–1.48)	*.8*	1.35 (1.02–1.78)	*2.1*	1,148	*20.5*	1.05 (.84–1.31)	*.4*	1.25 (.96–1.63)	*1.7*
Year group												
S2 (age 13 years)	807	*88.2*	1.00		1.00		806	*10.3*	1.00		1.00	
S3 (age 14 years)	787	*92.1*	1.52 (1.03–2.25)	*2.1*	1.51 (.99–2.27)	*2.0*	785	*18.2*	1.93 (1.38–2.71)	*3.9*	1.98 (1.39–2.81)	*3.9*
S4 (age 15 years)	753	*95.6*	2.99 (1.96–4.57)	*5.1*	2.86 (1.82–4.49)	*4.6*	751	*32.8*	4.32 (3.20–5.84)	*9.6*	4.82 (3.48–6.68)	*9.6*
Residential deprivation												
High status (low deprivation)	569	*90.7*	1.00		1.00		569	*11.8*	1.00		1.00	
Medium status	909	*92.7*	1.30 (.88–1.94)	*1.3*	1.20 (.81–1.77)	*.9*	906	*20.3*	1.88 (1.28–2.75)	*3.3*	1.75 (1.22–2.51)	*3.1*
Low status (high deprivation)	343	*91.2*	1.06 (.63–1.78)	*.2*	.83 (.51–1.37)	*−.7*	341	*26.1*	2.57 (1.68–3.95)	*4.4*	2.13 (1.39–3.26)	*3.5*
Missing	526	*92.2*	1.20 (.74–1.94)	*.8*	.97 (.61–1.54)	*−.1*	526	*25.1*	2.60 (1.78–3.80)	*5.0*	2.05 (1.43–2.94)	*4.0*
Family Affluence Scale												
High status (high affluence)	589	*91.3*	1.00		1.00		586	*19.8*	1.00		1.00	
Medium status	1,223	*92.2*	1.08 (.72–1.64)	*.4*	1.18 (.78–1.78)	*.8*	1,223	*19.6*	.96 (.73–1.26)	*−.3*	.97 (.73–1.28)	*−.2*
Low status (low affluence)	535	*91.8*	1.02 (.64–1.63)	*.1*	1.07 (.70–1.65)	*.3*	533	*21.8*	1.14 (.83–1.57)	*.8*	.95 (.68–1.32)	*−.3*
Subjective socioeconomic status												
High status	647	*91.0*	1.00		1.00		646	*17.5*	1.00		1.00	
Medium status	1,237	*92.3*	1.20 (.85–1.69)	*1.0*	1.21 (.83–1.77)	*1.0*	1,236	*20.2*	1.18 (.90–1.54)	*1.2*	1.19 (.89–1.59)	*1.2*
Low status	463	*92.0*	1.13 (.69–1.86)	*.5*	1.18 (.69–2.01)	*.6*	460	*23.7*	1.42 (1.04–1.92)	*2.3*	1.40 (.98–2.00)	*1.9*
SSS-peer												
High status	585	*95.7*	1.00		1.00		584	*34.8*	1.00		1.00	
Medium status	1,176	*92.2*	.51 (.34–.77)	*−3.2*	.52 (.34–.79)	*−3.1*	1,173	*17.0*	.38 (.29–.49)	*−7.4*	.35 (.26–.45)	*−7.7*
Low status	586	*87.5*	.29 (.19–.46)	*−5.4*	.26 (.16–.42)	*−5.6*	585	*11.8*	.25 (.18–.34)	*−8.5*	.19 (.13–.27)	*−9.1*
SSS-scholastic												
High status	585	*87.2*	1.00		1.00		583	*8.6*	1.00		1.00	
Medium status	1,182	*92.3*	1.79 (1.28–2.51)	*3.4*	1.81 (1.27–2.57)	*3.3*	1,181	*18.6*	2.45 (1.76–3.41)	*5.3*	2.55 (1.76–3.70)	*5.0*
Low status	580	*95.9*	3.44 (2.10–5.64)	*4.9*	3.42 (2.10–5.57)	*5.0*	578	*34.9*	5.84 (3.99–8.53)	*9.2*	5.79 (3.84–8.73)	*8.5*
SSS-sports												
High status	588	*91.3*	1.00		1.00		585	*13.7*	1.00		1.00	
Medium status	1,170	*92.0*	1.10 (.77–1.57)	*.5*	.97 (.67–1.41)	*−.2*	1,169	*20.4*	1.63 (1.25–2.11)	*3.7*	1.42 (1.05–1.93)	*2.3*
Low status	589	*92.4*	1.17 (.77–1.79)	*.8*	1.02 (.67–1.56)	*.1*	588	*26.0*	2.26 (1.65–3.10)	*5.1*	1.93 (1.34–2.78)	*3.6*
*N (% ever drinker/usually five or more drinks)*	*2,347*	*91.9*					*2,342*	*20.1*				

OR = odds ratio; CI = confidence interval; AOR = adjusted odds ratio; SSS = subjective social status.
