# Supplementary material for: Adolescent Socioeconomic and School-Based Social Status, Smoking, and Drinking
Source: J Adolesc Health. 2015 Jul;57(1):37–45. doi: 10.1016/j.jadohealth.2015.03.020 (PMC4510202; doi:10.1016/j.jadohealth.2015.03.020)
Supplement: Supplementary Table 1 [file mmc1.docx]

**SUPPLEMENTARY TABLE 1: Smoking and drinking measures according to SSS-peer excluding “trouble-maker”: (a) numbers (and row percentages); (b) crude odds ratios (and 95% confidence intervals).**

|  |  | | | |  | |  |  | | | |  | |
| --- | --- | --- | --- | --- | --- | --- | --- | --- | --- | --- | --- | --- | --- |
|  | **Ever-smoker** | | | | | |  | **Weekly smoker** | | | | | |
|  | **(a)**  **Ns (and row%)** | | | | **(b)**  **Crude ORs** | |  | **(a)**  **Ns (and row%)** | | | | **(b)**  **Crude ORs** | |
|  | **Never** | | **Ever** | |  |  |  | **No** | | **Yes** | |  |  |
|  | **N** | ***(row %)*** | **N** | ***(row %)*** | **OR (95% CI)** | ***t*** |  | **N** | ***(row %)*** | **N** | ***(row %)*** | **OR (95% CI)** | ***t*** |
| **Subjective Social Status - peer excluding “trouble-maker” *** |  |  |  |  |  |  |  |  |  |  |  |  |  |
| High status | 303 | *(59.4)* | 207 | *(40.6)* | 1.00 |  |  | 433 | *(85.1)* | 76 | *(14.9)* | 1.00 |  |
| Medium status | 851 | *(70.3)* | 360 | *(29.7)* | 0.62 (0.50-0.77) | *-4.4* |  | 1089 | *(89.9)* | 122 | *(10.1)* | 0.65 (0.49-0.87) | *-3.0* |
| Low status | 446 | *(71.4)* | 179 | *(28.6)* | 0.59 (0.44-0.77) | *-3.8* |  | 572 | *(91.5)* | 53 | *(8.5)* | 0.55 (0.38-0.80) | *-3.2* |
|  |  |  |  |  |  |  |  |  |  |  |  |  |  |
| *N* | *1600* | *(68.2)* | *746* | *(31.8)* | *2346* |  |  | *2094* | *(89.3)* | *251* | *(10.7)* | *2345* |  |
|  |  |  |  |  |  |  |  |  |  |  |  |  |  |
|  |  |  |  |  |  |  |  |  |  |  |  |  |  |
|  | **Ever-drinker** | | | | | |  | **Usually consume five or more drinks** | | | | | |
|  | **(a)**  **Ns (and row%)** | | | | **(b)**  **Crude ORs** | |  | **(a)**  **Ns (and row%)** | | | | **(b)**  **Crude ORs** | |
|  | **Never** | | **Ever** | |  |  |  | **No** | | **Yes** | |  |  |
|  | **N** | ***(row %)*** | **N** | ***(row %)*** | **OR (95% CI)** | ***t*** |  | **N** | ***(row %)*** | **N** | ***(row %)*** | **OR (95% CI)** | ***t*** |
| **Subjective Social Status - peer excluding “trouble-maker” *** |  |  |  |  |  |  |  |  |  |  |  |  |  |
| High status | 26 | *(5.1)* | 484 | *(94.9)* | 1.00 |  |  | 349 | *(68.6)* | 160 | *(31.4)* | 1.00 |  |
| Medium status | 92 | *(7.6)* | 1120 | *(92.4)* | 0.64 (0.40-1.02) | *-1.9* |  | 983 | *(81.3)* | 226 | *(18.7)* | 0.49 (0.38-0.64) | *-5.5* |
| Low status | 72 | *(11.5)* | 553 | *(88.5)* | 0.39 (0.25-0.61) | *-4.1* |  | 538 | *(86.2)* | 86 | *(13.8)* | 0.34 (0.25-0.48) | *-6.3* |
|  |  |  |  |  |  |  |  |  |  |  |  |  |  |
| *N* | *190* | *(8.1)* | *2157* | *(91.9)* | *2347* |  |  | *1870* | *(79.9)* | *472* | *(20.1)* | *2342* |  |
|  |  |  |  |  |  |  |  |  |  |  |  |  |  |

***** derived by summing responses to the “popular”; “powerful”; “respected” and “attractive or stylish” ladders
