# Supplementary material for: Adolescent Socioeconomic and School-Based Social Status, Smoking, and Drinking
Source: J Adolesc Health. 2015 Jul;57(1):37–45. doi: 10.1016/j.jadohealth.2015.03.020 (PMC4510202; doi:10.1016/j.jadohealth.2015.03.020)
Supplement: Supplementary Table 2 [file mmc2.docx]

**SUPPLEMENTARY TABLE 2: Ever and weekly smoker according to gender and status measures: unadjusted odds ratios (OR) with 95% confidence intervals (CI) for pupils from each year group and significance of interaction with year group.**

|  |  |  |  |  |  |  |  |  |  |
| --- | --- | --- | --- | --- | --- | --- | --- | --- | --- |
|  | **Ever-smoker** | | | |  | **Weekly smoker** | | | |
|  |  |  |  |  |  |  |  |  |  |
|  | **S2 pupils (age 13)**  **OR (%% CI)** | **S3 pupils (age 14)**  **OR (%% CI)** | **S4 pupils (age 15)**  **OR (95% CI)** | **Significance of interaction with year group** |  | **S2 pupils (age 13)**  **OR (%% CI)** | **S3 pupils (age 14)**  **OR (%% CI)** | **S4 pupils (age 15)**  **OR (95% CI)** | **Significance of interaction with year group** |
| **Gender** |  |  |  |  |  |  |  |  |  |
| Males | 1.00 | 1.00 | 1.00 |  |  | 1.00 | 1.00 | 1.00 |  |
| Females | 0.97 (0.66-1.42) | 1.06 (0.75-1.50) | 1.13 (0.80-1.61) | 0.728; 0.538 |  | 1.10 (0.60-2.01) | 0.57 (0.37-0.88) | 1.22 (0.75-1.98) | 0.079; 0.781 |
| **Residential deprivation** |  |  |  |  |  |  |  |  |  |
| High status (low deprivation) | 1.00 | 1.00 | 1.00 |  |  | 1.00 | 1.00 | 1.00 |  |
| Medium status | 4.98 (2.63-9.43) | 2.28 (1.46-3.53) | 1.08 (0.73-1.60) | 0.043; 0.000 |  | 4.33 (1.37-13.71) | 3.78 (1.40-10.22) | 1.61 (0.84-3.09) | 0.858; 0.133 |
| Low status (high deprivation) | 5.55 (2.80-11.00) | 3.99 (2.19-7.26) | 2.07 (1.13-3.80) | 0.465; 0.031 |  | 5.93 (1.43-24.60) | 7.73 (2.70-22.11) | 3.19 (1.45-7.03) | 0.763; 0.442 |
| Missing | 5.63 (3.00-10.58) | 2.34 (1.47-3.72) | 2.89 (1.83-4.55) | 0.025; 0.084 |  | 6.05 (1.61-22.73) | 4.83 (1.66-13.99) | 3.43 (1.84-6.39) | 0.788; 0.433 |
| **Family Affluence Scale** |  |  |  |  |  |  |  |  |  |
| High status (high affluence) | 1.00 | 1.00 | 1.00 |  |  | 1.00 | 1.00 | 1.00 |  |
| Medium status | 1.28 (0.80-2.05) | 1.22 (0.82-1.80) | 0.95 (0.66-1.36) | 0.877; 0.309 |  | 1.12 (0.47-2.64) | 1.14 (0.66-1.97) | 0.82 (0.55-1.23) | 0.966; 0.519 |
| Low status (low affluence) | 2.22 (1.38-3.56) | 1.33 (0.80-2.22) | 1.33 (0.84-2.10) | 0.140; 0.116 |  | 2.18 (0.91-5.24) | 2.15 (1.11-4.18) | 1.72 (1.04-2.84) | 0.977; 0.630 |
| **Subjective Socio-Economic Status** |  |  |  |  |  |  |  |  |  |
| High status | 1.00 | 1.00 | 1.00 |  |  | 1.00 | 1.00 | 1.00 |  |
| Medium status | 0.94 (0.61-1.46) | 1.06 (0.71-1.58) | 1.20 (0.80-1.78) | 0.704; 0.420 |  | 0.95 (0.48-1.88) | 0.75 (0.43-1.33) | 1.24 (0.78-1.97) | 0.592; 0.518 |
| Low status | 1.17 (0.73-1.89) | 1.64 (0.97-2.78) | 1.28 (0.81-2.04) | 0.336; 0.783 |  | 0.91 (0.43-1.94) | 1.40 (0.69-2.83) | 1.39 (0.70-2.77) | 0.404; 0.402 |
| **Subjective Social Status - peer** |  |  |  |  |  |  |  |  |  |
| High status | 1.00 | 1.00 | 1.00 |  |  | 1.00 | 1.00 | 1.00 |  |
| Medium status | 0.32 (0.19-0.53) | 0.48 (0.32-0.72) | 0.58 (0.38-0.87) | 0.195; 0.068 |  | 0.26 (0.13-0.52) | 0.28 (0.18-0.46) | 0.91 (0.56-1.47) | 0.829; 0.003 |
| Low status | 0.37(0.21-0.65) | 0.35 (0.23-0.55) | 0.50 (0.29-0.85) | 0.877; 0.444 |  | 0.21 (0.09-0.53) | 0.22 (0.11-0.41) | 0.73 (0.39-1.35) | 0.976; 0.025 |
| **Subjective Social Status - scholastic** |  |  |  |  |  |  |  |  |  |
| High status | 1.00 | 1.00 | 1.00 |  |  | 1.00 | 1.00 | 1.00 |  |
| Medium status | 3.50 (1.85-6.63) | 4.08 (2.41-6.88) | 2.90 (1.83-4.61) | 0.711; 0.628 |  | 1.49 (0.58-3.80) | 8.47 (1.99-36.06) | 3.11 (1.43-6.78) | 0.043; 0.222 |
| Low status | 12.15 (6.72-21.95) | 11.11 (6.57-18.76) | 8.02 (4.61-13.94) | 0.818; 0.301 |  | 7.66 (3.11-18.85) | 32.44 (7.45-141.27) | 9.43 (4.32-20.58) | 0.092; 0.724 |
| **Subjective Social Status - sports** |  |  |  |  |  |  |  |  |  |
| High status | 1.00 | 1.00 | 1.00 |  |  | 1.00 | 1.00 | 1.00 |  |
| Medium status | 1.48 (0.98-2.23) | 1.40 (0.94-2.06) | 1.70 (1.21-2.40) | 0.835; 0.593 |  | 2.44 (0.94-6.38) | 1.86 (0.96-3.61) | 2.04 (1.12-3.72) | 0.637; 0.747 |
| Low status | 1.64 (0.99-2.72) | 1.65 (1.04-2.62) | 2.47 (1.63-3.75) | 0.995; 0.210 |  | 2.54 (0.87-7.44) | 2.32 (1.02-5.28) | 2.92 (1.37-6.19) | 0.893; 0.830 |
|  |  |  |  |  |  |  |  |  |  |
| *N* | *807* | *786* | *753* | *2346* |  | *807* | *785* | *753* | *2345* |
|  |  |  |  |  |  |  |  |  |  |
