# Supplementary material for: Adolescent Socioeconomic and School-Based Social Status, Smoking, and Drinking
Source: J Adolesc Health. 2015 Jul;57(1):37–45. doi: 10.1016/j.jadohealth.2015.03.020 (PMC4510202; doi:10.1016/j.jadohealth.2015.03.020)
Supplement: Supplementary Table 3 [file mmc3.docx]

**SUPPLEMENTARY TABLE 3: Ever drinker and usually consume five or more drinks at a time according to gender and status measures: unadjusted odds ratios (OR) with 95% confidence intervals (CI) for pupils from each year group and significance of interaction with year group.**

|  |  |  |  |  |  |  |  |  |  |
| --- | --- | --- | --- | --- | --- | --- | --- | --- | --- |
|  | **Ever-drinker** | | | |  | **Usually consume five or more drinks** | | | |
|  |  |  |  |  |  |  |  |  |  |
|  | **S2 pupils (age 13)**  **OR (%% CI)** | **S3 pupils (age 14)**  **OR (%% CI)** | **S4 pupils (age 15)**  **OR (95% CI)** | **Significance of interaction with year group** |  | **S2 pupils (age 13)**  **OR (%% CI)** | **S3 pupils (age 14)**  **OR (%% CI)** | **S4 pupils (age 15)**  **OR (95% CI)** | **Significance of interaction with year group** |
| **Gender** |  |  |  |  |  |  |  |  |  |
| Males | 1.00 | 1.00 | 1.00 |  |  | 1.00 | 1.00 | 1.00 |  |
| Females | 0.90 (0.63-1.29) | 1.52 (0.83-2.78) | 1.23 (0.64-2.36) | 0.133; 0.393 |  | 0.97 (0.61-1.55) | 0.88 (0.61-1.26) | 1.28 (0.89-1.86) | 0.737; 0.341 |
| **Residential deprivation** |  |  |  |  |  |  |  |  |  |
| High status (low deprivation) | 1.00 | 1.00 | 1.00 |  |  | 1.00 | 1.00 | 1.00 |  |
| Medium status | 1.51 (0.94-2.42) | 1.02 (0.46-2.26) | 1.42 (0.51-3.94) | 0.394; 0.914 |  | 6.45 (1.79-23.20) | 2.27 (1.26-4.09) | 1.39 (0.85-2.25) | 0.136; 0.025 |
| Low status (high deprivation) | 1.06 (0.53-2.10) | 0.86 (0.33-2.26) | 1.25 (0.40-3.91) | 0.727; 0.802 |  | 11.15 (2.90-42.91) | 3.40 (1.89-6.10) | 1.50 (0.83-2.70) | 0.104; 0.006 |
| Missing | 1.71 (0.91-3.21) | 0.67 (0.28-1.61) | 1.38 (0.43-4.43) | 0.081; 0.750 |  | 7.33 (2.01-26.79) | 2.58 (1.32-5.05) | 2.41 (1.53-3.79) | 0.150; 0.103 |
| **Family Affluence Scale** |  |  |  |  |  |  |  |  |  |
| High status (high affluence) | 1.00 | 1.00 | 1.00 |  |  | 1.00 | 1.00 | 1.00 |  |
| Medium status | 1.51 (0.78-2.92) | 0.97 (0.55-1.71) | 0.79 (0.31-2.04) | 0.305; 0.260 |  | 1.35 (0.65-2.82) | 1.17 (0.71-1.94) | 0.88 (0.59-1.31) | 0.748; 0.300 |
| Low status (low affluence) | 1.55 (0.76-3.17) | 0.74 (0.36-1.51) | 0.81 (0.28-2.36) | 0.140; 0.312 |  | 1.89 (0.87-4.12) | 1.11 (0.61-2.05) | 1.09 0.69-1.70) | 0.279; 0.213 |
| **Subjective Socio-Economic Status** |  |  |  |  |  |  |  |  |  |
| High status | 1.00 | 1.00 | 1.00 |  |  | 1.00 | 1.00 | 1.00 |  |
| Medium status | 0.82 (0.51-1.32) | 1.64 (0.88-3.05) | 1.11 (0.41-3.03) | 0.076; 0.578 |  | 0.91 (0.56-1.47) | 0.89 (0.59-1.36) | 1.25 (0.77-2.03) | 0.967; 0.344 |
| Low status | 0.78 (0.39-1.55) | 1.15 (0.47-2.82) | 1.38 (0.40-4.76) | 0.487; 0.416 |  | 1.06 (0.52-2.18) | 1.26 (0.74-2.13) | 1.27 (0.76-2.12) | 0.705; 0.682 |
| **Subjective Social Status - peer** |  |  |  |  |  |  |  |  |  |
| High status | 1.00 | 1.00 | 1.00 |  |  | 1.00 | 1.00 | 1.00 |  |
| Medium status | 0.45 (0.26-0.77) | 0.94 (0.44-2.02) | 0.20 (0.04-0.93) | 0.115; 0.321 |  | 0.19 (0.10-0.37) | 0.32 (0.21-0.49) | 0.49 (0.33-0.72) | 0.211; 0.017 |
| Low status | 0.33 (0.19-0.58) | 0.31 (0.14-0.67) | 0.17 (0.03-0.87) | 0.866; 0.440 |  | 0.24 (0.12-0.47) | 0.24 (0.14-0.42) | 0.24 (0.14-0.40) | 0.978; 0.988 |
| **Subjective Social Status - scholastic** |  |  |  |  |  |  |  |  |  |
| High status | 1.00 | 1.00 | 1.00 |  |  | 1.00 | 1.00 | 1.00 |  |
| Medium status | 1.70 (1.10-2.64) | 1.38 (0.71-2.71) | 2.50 (1.07-5.85) | 0.601; 0.420 |  | 1.16 (0.57-2.33) | 2.91 (1.57-5.40) | 2.76 (1.65-4.61) | 0.047; 0.045 |
| Low status | 4.44 (2.12-9.30) | 2.51 (0.97-6.52) | 3.00 (1.07-8.37) | 0.341; 0.531 |  | 4.92 (2.52-9.62) | 6.66 (3.35-13.23) | 6.20 (3.32-11.60) | 0.525; 0.611 |
| **Subjective Social Status - sports** |  |  |  |  |  |  |  |  |  |
| High status | 1.00 | 1.00 | 1.00 |  |  | 1.00 | 1.00 | 1.00 |  |
| Medium status | 1.04 (0.58-1.86) | 1.16 (0.64-2.09) | 1.14 (0.54-2.40) | 0.792; 0.841 |  | 1.34 (0.80-2.26) | 1.32 (0.77-2.26) | 2.19 (1.51-3.19) | 0.963; 0.125 |
| Low status | 0.67 (0.37-1.19) | 1.42 (0.65-3.09) | 2.30 (0.82-6.50) | 0.120; 0.037 |  | 1.62 (0.76-3.47) | 1.82 (1.02-3.26) | 2.58 (1.61-4.13) | 0.807; 0.295 |
|  |  |  |  |  |  |  |  |  |  |
| *N* | *807* | *787* | *753* | *2347* |  | *806* | *785* | *751* | *2342* |
|  |  |  |  |  |  |  |  |  |  |
